# Epidemiology of Childhood Type 1 Diabetes Mellitus in Nile Delta, Northern Egypt - A Retrospective Study

**DOI:** 10.4274/Jcrpe.1171

**Published:** 2014-03-05

**Authors:** Magdy Abd El-Monem El-Ziny, Nanees Abdel-Badie Salem, Amany Kamal El-Hawary, Nehad Mohamed Chalaby, Ashraf Abd-Elmoneim Elsharkawy

**Affiliations:** 1 Mansoura University Children’s Hospital, Pediatric Endocrinology and Diabetes Unit, Mansoura, Egypt

**Keywords:** type 1 diabetes mellitus, Egypt, Epidemiology

## Abstract

**Ob­jec­ti­ve**: The geographical incidence of type 1 diabetes mellitus (T1DM) varies widely worldwide. Both genetic and environmental factors have been implicated, although environmental factors are still speculative and elusive. More epidemiological studies are needed to uncover such factors. To date, there are no reported studies on the epidemiology of childhood T1DM in Nile Delta, Egypt. We aimed to define the incidence, prevalence and demographic characteristics of T1DM in children (0-18 years) living in the Nile Delta region, one of the most densely populated areas in Egypt.

**Methods**: The study included all T1DM patients aged 0-18 years who lived in the Nile Delta region of Egypt and who were either diagnosed at or referred to Mansoura University Children’s Hospital (MUCH) between 1 January 1994 and 31 December 2011. The hospital files of the patients were reviewed. General population data on the 0-18 year age group in the Nile Delta governorates were also presented.

**Results**: From a total of 1600 T1DM patients, 891 (55.7%) were females (p=0.000) and 935 (58.4%) were from rural areas (p=0.000). Calculated age-adjusted incidence of T1DM in 1996, 2006 and 2011 were 0.7, 2.0 and 3.1/10^5^/year, respectively, while calculated age-adjusted prevalence of T1DM in the same years were 1.9, 15.5 and 26.8/10^5^/year, respectively. Patients presented most frequently in the 5-10 year age group (p<0.000) and in winter months (p=0.009).

**Conclusion**: In this first childhood T1DM epidemiology study in the Nile Delta region of Egypt, T1DM incidence and prevalence were found to show an increase over the past 18 years (1994-2011). Incidence and prevalence were higher in females and more cases were found to originate from rural areas.

## INTRODUCTION

Type 1 diabetes mellitus (T1DM) is one of the most common endocrine and metabolic conditions in childhood. Data on incidence of childhood onset T1DM are very limited. Data from large epidemiological studies worldwide indicate that on an annual basis, the overall increase in the incidence of T1DM is around 3% and about 78 000 children under age 15 years develop T1DM worldwide (1).

The worldwide geographic variation in the incidence of T1DM is striking. The overall standardized incidence varies from 0.1/100 000 per year in the Zunyi region within China to more than 40/100 000 per year in Finland in children under the age of 15 years (2,3). This almost 400-fold variation in incidence can hardly be explained by genetic factors alone. Environmental factors have long been implicated in the pathogenesis of T1DM both as initiator and potentiators of pancreatic β-cells destruction (4).

Among Eastern Mediterranean and Middle Eastern countries, the largest contribution to the total number of estimated childhood T1DM cases comes from Egypt which accounts for about a quarter of the region’s total. The incidence varies between 1/100 000 per year (Pakistan) and 8/100 000 per year (Egypt) in children under the age of 15 years (5).

**Figure 1:**
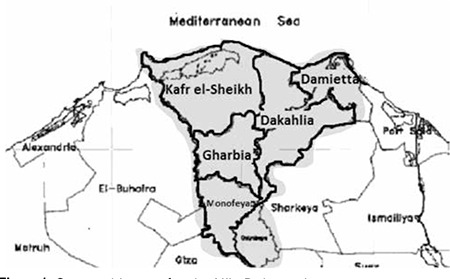
Geographic map for the Nile Delta region Area of the study (Dakahlia, Damietta, Kafr El-Sheikh, Gharbia and other governorates)

Egypt is located on the northeastern corner of Africa and has the largest settled population among the Arab countries. The Nile Delta is one of the most densely populated and cultivated regions in Egypt. Egypt is divided into 28 governorates, nine of them located in the Nile Delta including, Dakahlia, Damietta, Kafr el-Sheikh and Gharbia; each is subdivided into urban and rural areas (Figure 1) (6).

Epidemiological studies for childhood T1DM from Egypt are scarce. This has been attributed to many reasons including lack of diabetes registries, scattered medical facilities and suboptimal capturing of new cases. To the best of our knowledge, there are no reported epidemiological data for childhood onset T1DM in the Nile Delta region. This study is designed to define the incidence, prevalence and demographic characteristics of T1DM in children aged 0-18 years between 1994 and 2011 in an Egyptian subpopulation living in the Nile Delta region, one of the most densely populated areas in Egypt, aiming for a better understanding of the risk factors as well as to plan future strategies to control this disease.

## METHODS

Mansoura University Children’s Hospital (MUCH) is a tertiary pediatric center in the Nile Delta region which has an Endocrine and Diabetes unit established in 1994. This unit provides medical care to all children with T1DM who are under 18 years of age and reside in Dakahlia, Damietta and Kafr el-Sheikh governorates and to all children with T1DM from Gharbia and other governorates in the Nile Delta region who are referred to the unit (Figure 1).

Since the possibility that a child with T1DM could have been treated without hospitalization in Egypt must be regarded as exceptional, it was assumed that two sources could provide us with information in the present study, namely, the hospital files and pediatric diabetes clinic records of all children with T1DM who were diagnosed at or referred to MUCH between 1 January 1994 and 31 December 2011. Moreover, we reviewed the medical records of all children with a diagnosis of T1DM who are beneficiaries from Health Insurance services (almost all diabetic patients) under supervision of staff member of Endocrine and Diabetes unit of MUCH. The secondary source of validation was established through the review of T1DM patients’ basic data (name, surname, date and place of birth) to avoid multiple reports of the same patient.

The diagnosis of T1DM was made according to the World Helath Organization (WHO) criteria (7). In addition, we collected data on age at onset, sex, residency, month and year of diagnosis and distribution of patients among the Nile Delta governorates.

**Figure 2:**
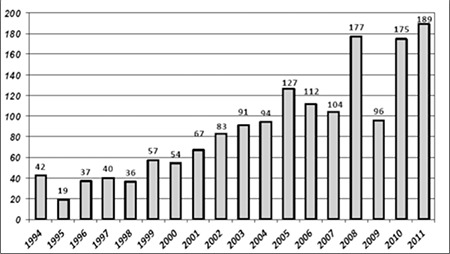
Annual numbers of new patients with type 1 diabetes mellitus (T1DM) among children aged 0-18 years in the Nile Delta region (1994-2011)

All Egyptian children <18 years of age residing in the Nile Delta region with new-onset T1DM diagnosed between 1994-2011 were included in the study. We excluded children with T2DM and those with diabetes secondary to post-surgical pancreatectomy, cystic fibrosis, or steroid therapy. Calculated age-adjusted incidence and prevalence rates were estimated and expressed per 100 000 population. Annual numbers of newly diagnosed patients were determined (Figure 2) and used as the numerator. General population data pertaining to the Nile Delta governorates and specifically to Dakahlia, Damietta, Kafr el-Sheikh and Gharbia in census years 1996 (8), 2006 (8) and 2011 (9) were obtained. The percentage and the number of the population <18 years were obtained (10) and used as the denominator.

The patients were divided into four age groups as: infancy and toddlerhood group (0-2 years), preschool group (3-5 years), middle childhood group (6-10 years) and adolescence group (11-18 years) (11). The months at which T1DM clinical diagnosis was made were grouped into seasons in order to investigate the possibility of seasonality in the initial presentation of T1DM.

The study was approved by the ethics committee of the Pediatric Department at MUCH. Statistical Analysis SPSS 16.0 for Windows® software package program (SPSS Inc., Chicago, IL.) was used in the analysis. Data were expressed as frequencies [n (%)]. Chi-square “chi2” test was used in comparing the data. A p-value smaller than 0.05 was accepted as statistically significant.

## RESULTS

A diagnosis of T1DM was made in a total of 1600 patients in the 0-18 year age group at MUCH over a period of 18 years starting on 1 January 1994 and ending on 31 December 2011. Annual numbers of diagnosed cases were found to increase slowly over many years, reaching highest levels in 2008, 2010 and 2011 (Figure 2). The median age at T1DM diagnosis was recorded at 12 and 10 years in females and males, respectively. All patients originated from the Nile Delta governorates at Northern Egypt. The group included 1162 patient from Dakahlia (72.6%), 175 from Damietta (10.9%), 50 from Kafr el-Sheikh (3.12%), 155 from Gharbia (9.6%) and 58 patients were from other governorates (3.78%).

Calculated age-adjusted incidence and prevalence rates of T1DM/10^5^ population within the 0-18 year age group in the studied governorates of the Nile Delta region are shown in Table 1 and Figure 3. Distribution of T1DM patients according to age groups, gender, residency and seasonality at time of T1DM diagnosis are shown in Table 2.

The frequency of T1DM diagnosis significantly increased with age, reaching a peak at age group 6-10 years (p=0.000), before falling to a much lower rate at age group 11-18 years. This trend was evident in the total patient population as well as in each gender (Table 2).

Total numbers for female and male patients were 891 (55.7%) and 709 (44.3%), respectively and the female/male ratio was 1.3 in favor of girls. This significant female predominance was evident in the total patient population (891 vs. 709, p=0.000) and also in patients from both rural (520 vs. 415, p=0.001) and urban (371 vs. 294, p=0.003) areas. In addition, female predominance was present in all age groups, but this difference was statistically significant only in the age group 6-10 years (p=0.000) (Table 2).

Of a total of 1600 patients, 935 were from rural areas of the Nile Delta region and 665 were from its urban areas. This higher rate of T1DM in rural compared to urban areas was statistically significant in the total patient population and in each gender separately (p=0.000) (Table 2).

The percentage of distribution of cases by month of T1DM diagnosis is shown in Table 3. There was no single month with a significantly higher observed rate of T1DM diagnosis, but between November and March, the incidence remained relatively stable and significantly high among the total patient population, separately for each gender and also in both urban and rural areas (p=0.000), in addition to a peak in July in urban areas and male patients. However, May was the only month for which the rate of T1DM diagnosis was significantly low (Table 3).

When the total patient population and both genders were examined according to the season at T1DM diagnosis, the most frequent time of presentation was in the winter followed by autumn (p=0.000) (Table 2). Significant female predominance was observed in the winter (p=0.009) and in the spring (p=0.002) (Table 2). On examination of the impact of seasonality on different age groups, we found a significant seasonality only in age group 6-10 years with a higher observed rate of T1DM diagnosis in winter (276 patients), followed by autumn (215 patients), summer (196 patients) and lowest rate in spring (179 patients) (p=0.000). No significant seasonality was detected in age group 0-2 years (p=0.694), age group 3-5 years (p=0.774) or in age group 11-18 years (p=0.211).

## DISCUSSION

Childhood-onset T1DM is a serious, debilitating disease, with life-threatening complications, in addition to its health provision and resource implications. The present study enrolled 1600 patients in the 0-18 years age group with T1DM over a period of 18 years (1994-2011) in the Nile Delta region, which is one of the most densely populated and cultivated regions in Egypt. Calculated age-adjusted T1DM incidence in 1996, 2006 and 2011 was 0.7, 2.0 and 3.1/10^5^/year, respectively, while age-adjusted T1DM prevalence in the same years was 1.9, 15.5 and 26.8/10^5^/year, respectively (Table 1).

This study demonstrates that childhood T1DM incidence in the Nile Delta region of Egypt is similar to that in the “low incidence” category of the WHO Diabetes Mondiale (WHO DiaMond) project classification (2).

In Egypt, epidemiological studies for childhood T1DM are scarce. The prevalence of T1DM was estimated at 109/10^5^ and 112/10^5^ in children of school age in the Heliopolis (12) and El-Manial districts (13) of Cairo, respectively.

In Africa and Middle East, data regarding the epidemiology of childhood-onset T1DM are also sparse. The incidence rates in our pediatric population from the Nile Delta region were lower than those reported from neighboring countries including Sudan (10.1/10^5^) (14), Libya (7.8/10^5^) (15), Tunisia (6.76/10^5^) (16), Saudi Arabia (27.5/10^5^) (17), Kuwait (20.1/10^5^) (18) and Turkey (7.2/10^5^) (19) (Table 4).

The geographical variability in the incidence of T1DM may be explained by genetic variations. It is well-established that genetic factors, notably the HLA system, influence the susceptibility to T1DM (20); however, the increases in T1DM disease incidence observed in many countries in recent years cannot be explained by genetic factors alone (21) and even among ethnically similar populations, T1DM incidence can vary (22), a finding which highlights the role of the environment in disease evolution.

The low incidence of T1DM in the current study can be explained by the fact that the Egyptian population resides in areas with plentiful and tolerable exposure to sunlight throughout the year which permits longtime outdoor activities. This theory is supported by the reported negative association between average daily ultraviolet B radiation (UVB) exposure and subsequent endogenous vitamin D production and the temporal incidence of T1DM (23). However, the finding in Sardinia -Italy’s high incidence of T1DM despite high levels of UVB exposure- is not in accordance with this theory (24).

The incidence of childhood T1DM is not uniform at all ages. In most registries, the typical pattern of T1DM occurrence by age showed that the incidence increases with age and peaks generally in the peripubertal period with the associated gender effect which starts 1-2 years earlier in girls compared to boys (25). In children under age 15, the DiaMond Project Group reported a higher risk of developing T1DM in the 10-14 year age group, while the age group 5-9 years had a medium risk and the age group 0-4 years had a lower risk. The age group 10-14 years had about twice the risk of developing T1DM compared to children younger than 5 years and this trend did not vary by gender (2). However, this is not a consistent observation since an increased incidence of childhood T1DM in the age group younger than 5 years compared to the older age groups has been reported in a multicenter study for childhood T1DM in Europe (26).

In our study population, more patients presented at 12 and 10 years of age for females and males, respectively. In the total patient population and also in both genders, T1DM occurrence increased significantly with age, reaching a peak in the age group 6-10 years, before falling to a much lower rate in the age group 11-18 years.

However, data from Tunisia (16), Kuwait (18) and Turkey (males only) (19) showed that the incidence of T1DM peaked in the age group 10-14 years and peaked in 5-9 years in Turkish females only (19) (Table 4).

Although boys and girls in general have a similar risk of T1DM under age 15 (27), some reports from Europe suggest a female predominance in lower-risk populations and slight male excess in the high-risk groups (28). In a recent data analysis, more males were found to develop T1DM at younger ages, while females predominated during the peripubertal period (29).

The current study showed a significant female predominance among the total patient population in both rural and urban areas, but only in the age group 6-10 years. Our results appear to be in line with literature data where female predominance was significant among Libyan (15), Saudi (17) and Turkish (19) T1DM patients. Only a slight and not significant higher incidence of T1DM in Sudanese females (14) and Kuwaiti males (18) was reported, while no gender difference was observed among Tunisians (16) (Table 4).

In the literature, there is conflicting evidence as to whether the prevalence of T1DM is lower (19,30) or higher (31,32) in children living in rural areas. We assume that specific environmental and sociodemographic factors (e.g. changing life style), not yet identified, vary between urban and rural areas and probably explain these differences in the prevalence (19,30,31,32).

In the present study, the significantly higher T1DM occurrence in rural areas can possibly be attributed to accidental exposure of children living in these areas to certain environmental β-cell toxins such as organophosphorus compounds (OPCs) including insecticides, rodenticides (Vacor) and pediculicides (malathion). This explanation is supported by the reported epidemiological evidence that exposure to certain environmental chemicals is implicated in T1DM pathogenesis through direct β-cells destruction or initiation of T1DM autoimmunity in humans (33,34).

In a recent preliminary study conducted for the first time in pediatric patients (age group 1.2-10 years) at our unit for evaluation of the possible association between T1DM in children and exposure to pesticides, the authors demonstrated measurable levels of several pesticide residues in the sera of newly diagnosed T1DM Egyptian children and malathion was reported as the most prevalent pesticide encountered (35).

In the present study, the rate of T1DM diagnosis was relatively high and stable between the months of November and March (corresponding to winter and early spring). In addition, a peak was observed in July in urban areas in male patients. The lowest rate of newly diagnosed T1DM children was recorded in May. The month of May is the beginning of the summer holidays and the lack of school stress could provide an explanation for this low rate. However, we cannot explain the peak of T1DM occurrence observed in male urban boys. Seasonality in T1DM diagnosis has been extensively studied, but the results are conflicting. Some studies have found evidence for seasonality (36,37), while others have not (38,39). Seasonality in T1DM diagnosis was confirmed in our study among the total patient population and in both genders separately during the winter-autumn period. However, this pattern of seasonality was evident only in the age group 6-10 years and female predominance was observed only in winter and spring. Our result appears to be in line with the results of studies reported among Sudanese (14), Libyan (15), Tunisian (16), Saudi (17) and Kuwaiti (18) children where more new cases were diagnosed as T1DM in the cooler months of the year. In contrast to our observations, older age groups and males appear to be more prone to exhibit seasonality in T1DM occurrence in some studies (40,41). Different suggestions were made to explain seasonality in T1DM diagnosis; seasonal viral infections (e.g. enteroviruses, rotavirus, mumps, rubella, cytomegalovirus) have been implicated in the etiology of T1DM (42,43,44). Higher food intake and less exercise have also been suggested to play a precipitating role in T1DM occurrence in winter months (45).

In conclusion, the results of the present study indicate that T1DM incidence and prevalence showed a progressive increase over a period of 18 years among children aged from 0 to 18 years living in the Nile Delta region. Higher T1DM occurrence was observed in rural areas and female predominance was evident. Seasonality in T1DM diagnosis was documented with a peak occurring in winter. Our observations confirm the need to develop a national registry for T1DM and the need for further multicenter epidemiological research studies covering the whole country to define the nationwide T1DM incidence and the related health data in Egypt.

## Figures and Tables

**Table 1:**
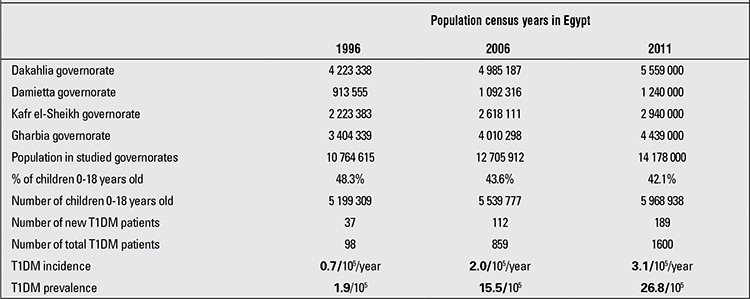
Calculated age-adjusted population size, incidence and prevalence rates of type 1 diabetes mellitus (T1DM) in Egyptian children (0-18 years) in the Nile Delta region in 1996, 2006 and 2011

**Table 2:**
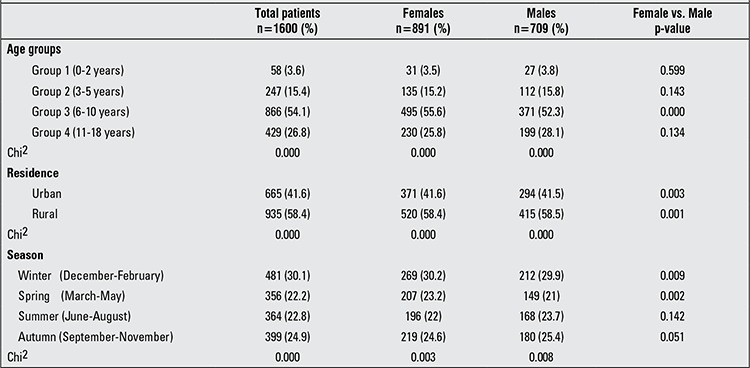
Demographic characteristics of type 1 diabetes mellitus (T1DM) patients

**Table 3:**
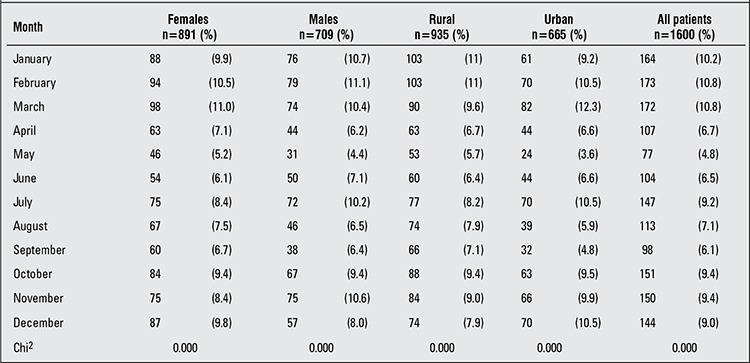
Distribution of type 1 diabetes mellitus (T1DM) patients by month at diagnosis

**Table 4:**
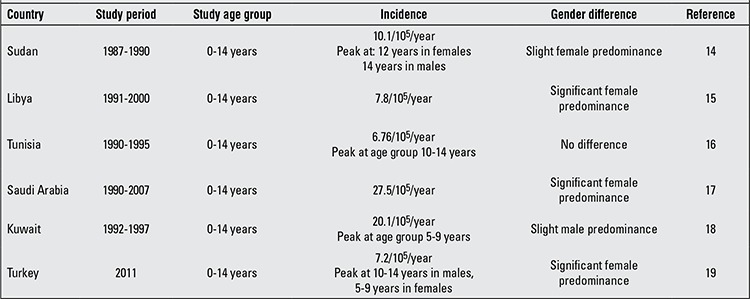
Incidence of type 1 diabetes mellitus (T1DM) in children in the neighboring countries in North Africa, Middle East, Turkey

**Figure 4:**
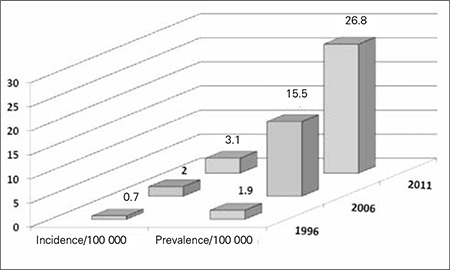
Calculated age-adjusted incidence and prevalence rates of type 1 diabetes mellitus (T1DM)/100 000 Egyptian children aged 0-18 years in 1996, 2006 and 2011
